# Vector Surveillance and Pathogen Detection in the Working Areas of Military Working Dogs in Eastern Austria

**DOI:** 10.3390/pathogens11050506

**Published:** 2022-04-24

**Authors:** Bernhard W. Sonnberger, Licha N. Wortha, Dietmar Rackl, Adelheid G. Obwaller, Anja Joachim, Hans-Peter Fuehrer

**Affiliations:** 1 Department of Pathobiology, Institute of Parasitology, University of Veterinary Medicine, 1210 Vienna, Austria; bw.sonnberger@gmail.com (B.W.S.); licha.wortha@vetmeduni.ac.at (L.N.W.); anja.joachim@vetmeduni.ac.at (A.J.); 2 Office of the State Government of Upper Austria, Department of Food Security and Veterinary Affairs, Directorate Social Affairs and Health, 4021 Linz, Austria; 3 Veterinary Service, Joint Support Command, Federal Ministry of Defense, 1120 Vienna, Austria; dietmar.rackl@bmlv.gv.at; 4 Research and Development, Division of Science, Federal Ministry of Defense, 1090 Vienna, Austria; adelheid.obwaller@bmlv.gv.at

**Keywords:** ticks, mosquitoes, surveillance, *Borrelia*, *Bartonella*, *Rickettsia*, *Babesia microti*

## Abstract

Vector-borne diseases play a major role in human and veterinary medicine worldwide. A previous study detected asymptomatic vector-borne pathogens in military working dogs stationed at a military base in eastern Austria, and a follow-up survey of potential arthropod vectors was conducted in spring 2019 and 2020 in the vicinity of the base to evaluate the presence of vectors and their carrier status for a range of canine and zoonotic pathogens. A total of 1324 ticks (nymphs and adults of *Ixodes ricinus*, comprising 92.9% of the collected specimens, and adults of *Haemaphysalis inermis*, a tick previously only rarely described in Austria, *Haemaphysalis concinna*, and *Dermacentor reticulatus*) were collected by flagging. In 44.1% (125/284) of all pools (n = 284), one infectious agent was found; in 27.8% (79/284) and in 1.1% (3/284), two and three different agents, respectively, could be identified. Overall, 72.9% of the pools contained at least one pathogen (*Borrelia* spp., *Rickettsia* spp., *Bartonella* spp., and *Babesia microti*). *Borrelia mijamotoi, B. lustinaniae*, and *B. microti* were previously only described in single cases in Austria. Mosquitoes were collected with BG-Sentinel traps monthly during the summer of 2019. A total of 71 individuals from 11 species were collected. No filarioid DNA was detected in the mosquito sample pools, although *Dirofilaria repens* had been present in the dogs from the military site. In conclusion, vector surveillance should be combined with the surveillance of an exposed population whenever possible to estimate the infection risks for dogs and their handlers.

## 1. Introduction

Vector-borne diseases play a major role worldwide in both human and veterinary medicine, and are frequently emerging in new areas around the world due to the spread of their vector species [1,2]. The most common ticks in Central Europe, including Austria, are *Ixodes ricinus, Ixodes canisuga, Ixodes hexagonus, Dermacentor reticulatus*, and *Haemaphysalis concinna* [3,4,5]. *Ixodes ricinus* is not only the most common tick species (even in dogs) in Central Europe, but also the vector of numerous pathogens such as *Anaplasma phagocytophilum, Borrelia* spp., *Rickettsia* spp., tick-borne encephalitis virus, and other, less common pathogens such as *Francisella tularensis* and, potentially, *Coxiella burnetii*, as well as members of the genus *Babesia* spp. [6,7,8,9]. *Dermacentorreticulatus* is, most notably, a carrier of *Babesia canis*, the causative agent of canine babesiosis [7]. The occurrence of babesiosis in dogs is directly linked to the occurrence of the tick *D. reticulatus*, which prefers cooler regions and is thus relatively common in Central Europe, including Austria [4,10]. Recent publications have also raised concerns that tick species previously not endemic to Austria may become established. This concerns *Hyalomma marginatum* in particular, but it might also be true for other species [11,12,13]. The introduction of new pathogens to Austria has also been reported. *Rickettsia raoultii* and *Borrelia miyamotoi* were recently detected in Austria for the first time in ticks and, subsequently, in humans [14,15].

Mosquito species in Austria can act as competent vectors for various pathogens, including *Dirofilaria repens*, a zoonotic parasite of carnivores, and it is assumed that other vector-competent species will also occur in Austria due to climate change or accidental import [16,17,18]. The first autochthonous human case of *D. repens* in Austria was described in 2006 [19], and the first finding of *D. repens* in Austrian mosquitoes was recorded in 2012 [20].

A study on vector-borne diseases in military dogs at the Military Working Dog Training Centre (MWDTC) in Kaisersteinbruch, Burgenland, Austria, detected several infections of *B. burgdorferi* sensu lato, *B. canis*, and *D. repens* in the examined dogs. These dogs had never worked abroad and had spent much of their lives at the MWDTC (although not all were born there) [21]. To confirm the local transmission of these (and possibly other) vector-transmitted pathogens, a follow-up study was conducted in the surrounding area of the MWDTC. The centre and the exercise and training areas of the dogs were located in an area where *I. ricinus* and also *D. reticulatus*, the vector not only of *B. canis* but also of *Rickettsia raoultii*, had already been previously detected [4,22]. It was assumed that *B. canis*, *B. burdorferi* s.l., and *D. repens* occurred in their corresponding vectors in the investigated area. Consequently, the mosquito and tick population surrounding the MWDTC was investigated during two summer seasons—2019 and 2020—for competent vector species, including human and veterinary pathogens, to specifically evaluate the risk of infection for dogs and their trainers.

## 2. Results

### 2.1. Ticks

**Figure 1:**
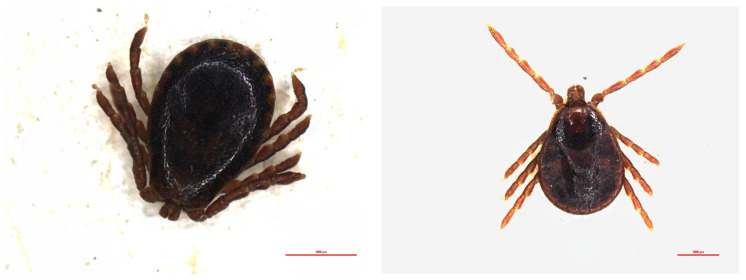
Adults of *Haemaphysalis inermis* (**left**: male, **right**: female).

In total, 1324 ticks (nymphs and adults, but no larvae) were collected and assigned to 284 pools. Four different species could be identified. The most frequent species was *I. ricinus* (92.9%; n = 1230), followed by *Haemaphysalis inermis* (3.6%; n = 48), *D. reticulatus* (3.1%; n = 41), and *Haemaphysalis concinna* (0.4%; n = 5). Sixty percent of the collected *I. ricinus* were nymphs while, of the other species, only adult individuals were found. A preponderance of female individuals could be observed, which was particularly evident in *H. inermis* (Figure 1). With the exception of *H. concinna*, which was not found in the shelterbelt, all tick species were collected from all three sites. In the forest, however, the finds of *H. inermis* and *D. reticulatus* were only sporadic.

#### Pathogen Detection in the Ticks

**Figure 2:**
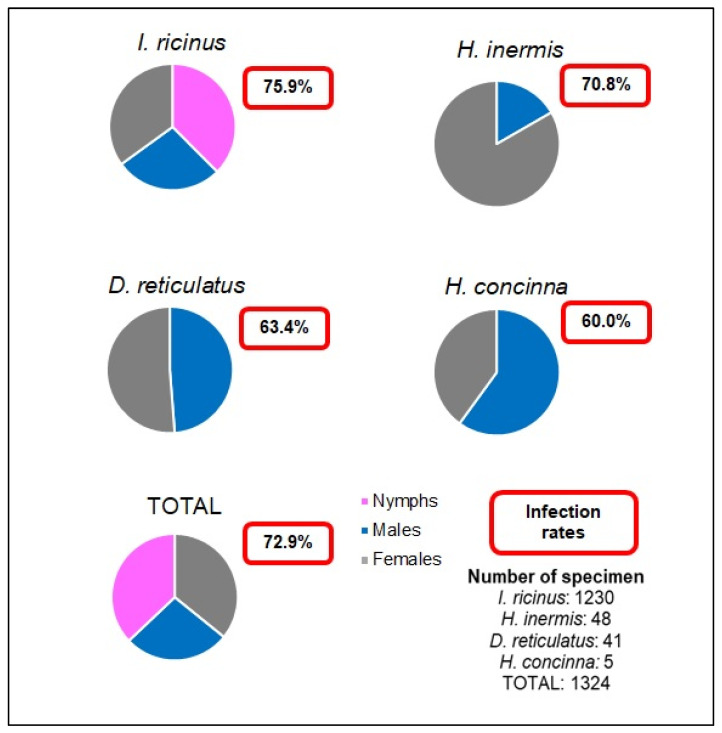
Collected ticks by species, stage, and infection rate.

In 44.1% of all pools (n = 284), one infectious agent was found; in 27.8% (79/284), two agents were found; and in 1.1% (3/284), three different agents could be identified (Table 1). Overall, 72.9% of the tick pools contained carriers of at least one agent, including the tick pools positive for *Bartonella* spp. and *Rickettsia* spp., though no conclusive classification to pathogen or endosymbiont could be made. Pools of *I. ricinus* (adults and nymphs) had an infection rate of 75.9%. Considering the Minimal Infection Rate (MIR), at least 11.9/100 *I. ricinus* were positive for at least one pathogen. The infection rates for *D. reticulatus* (63.4%), *H. inermis* (70.8%), and *H. concinna* (60.0%) were only slightly lower (Figure 2).

DNA of *Borrelia* spp. could be detected in *I. ricinus,* where it was found in 52.6% of the pools. Considering the MIR, at least 8.3/100 ticks were positive for *Borrelia* spp. The maximum likelihood estimation (MLE) returned an expected infection rate of 0.86 among the adult ticks and 0.99 among the nymphs. Considering only *Borrelia* of the *B. burgdorferi* s. l. complex (excluding *B. miyamotoi)*, 45.26% of the *I. ricinus* pools were positive, resulting in an MIR of 7.18%. Only 31.3% of the adults were positive for *Borrelia* spp., while the nymphs showed a much higher infection rate of 84.6%, but it should be mentioned that the number of individuals per pool of the nymphs was higher. In total, six different species and one undefined species, as well as eleven different strains of *Borrelia* spp., could be detected (Table 1).

Three different *Rickettsia* species could be unequivocally determined. *Rickettsia helvetica* was detected in 94 pools (51.1%) of *I. ricinus*, resulting in an MIR of 7.9/100 ticks. Sixty-one percent of *D. reticulatus* were PCR-positive for *R. raoultii*, and 64.6% of *H. inermis* were positive for *Rickettsia* sp. (Table 1).

Three different sequence types were detected, which may indicate different *Bartonella* species or strains within the same species. In total, 37 different sequences (accession numbers OL690436-OL690472) were determined. All tick species were positive at least once. The DNA of *Arsenophonus* sp. was detected by the *Bartonella* PCR protocol in one male pool, two female pools, and four nymph pools of *I. ricinus*. *Babesia microti* genotype 1 could be detected in one pool of female *I. ricinus* and one pool of *I. ricinus* nymphs. This resulted in an MIR of 0.17/100 ticks (Table 1).

### 2.2. Mosquitoes

**Figure 3:**
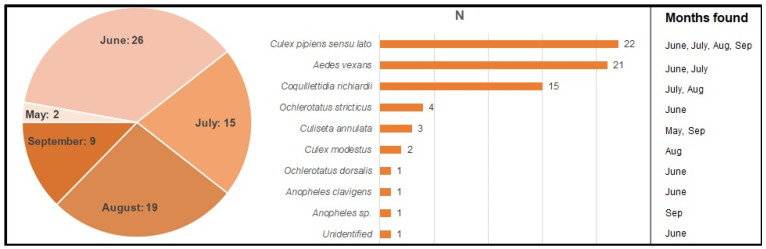
Number and species distribution of collected mosquitoes by collection month.

The mosquitoes were divided by species and the month of collection, resulting in a total of 17 pools. The number of individuals per pool varied from 1 to 19 mosquitoes. In total, 71 individuals of 11 species were found (Figure 3).

#### Pathogen Detection in the Mosquitoes

No filarioid DNA could be detected in any of the mosquito pools by PCR.

## 3. Discussion

The present study showed the occurrence of at least four different tick species in the collection area. While *I. ricinus*, *H. concinna*, and *D. reticulatus* are already well described in this part of Austria [4,23], the detection of *H. inermis* in the studied area can be highlighted as being of particular interest: although its occurrence in Austria has been mentioned in previous works [3,11,24], no recent reports have been made. Generally, *H. inermis* can be found in eastern parts of Europe and is listed as a vector of the potential human pathogen *R. helvetica* [25]. Recently, *B. miyamotoi* was also detected in *H. inermis* in Slovakia, a neighbouring country of Austria [26]. *D. reticulatus,* a vector of *B. canis*, could also be detected. This is of particular interest since *B. canis* has previously been found in military dogs in this area [21]; however, no DNA of *B. canis* was detected in the current catch.

After the first finding of *B. miyamotoi* in ticks in 2015, the detection in this study is only the second documentation of this pathogen in Austria, confirming the results from 2015 [14]. In particular, concerning the recent publication of autochthonous human infections with the associated hard tick relapsing fever, the results from the recent study are of current concern [15]. Furthermore, species of the *Borrelia burgdorferi* s. l. complex were detected. In detail, these were *B. afzelii*, *B. valaisiana*, *B. garinii*, *B. lusitaniae*, and *B. burgdorferi* sensu stricto (s. s.). *B. lusitaniae* was recently reported for the first time in Austria [27]. *Borrelia afzelii* and *B. garinii*, but occasionally also *B. lustinaniae* and *B. valaisiana*, can cause serious illnesses such as Lyme disease in humans [28,29]. *Borrelia burgdorferi* s. s. can cause Lyme disease in both humans and dogs [30]. Considering only the *B. burgdorgeri* s. l. complex, 45.3% of the *I. ricinus* pools were positive (MIR 7.18%). A meta-analysis from 2017 defined the mean prevalence as 19.3% for all *I. ricinus* (including nymphs and adults) [31]. Compared to these studies, the prevalence in the present study was slightly higher. In another area in Austria, the prevalence was 25.7% in ticks collected during 2002–2003 [32]. The data from this present work and the fact that both dogs and dog handlers do much of their work outdoors may indicate a significant risk for *Borrelia*-associated diseases in this work field. This is also shown by the results of the blood test of the military dogs, of which 10.6% were positive for specific antibodies against *B. burgdorferi* s. l. [21].

*Rickettsia raoultii* could be detected in 61.0% of the collected and examined *D. reticulatus*. The first detection of *R. raoultii* in *D. reticulatus* ticks in Austria was in 2016 [22]. In contrast to the present study, in a previous study, tick pools were used and an MIR of 14.9% was elaborated. Results similar to those in the present study were found, with 56.7% of *D. reticulatus* ticks in Germany and Poland being positive for *R. raoultii* [33,34]. In these studies, individual ticks were examined; therefore, the results can be compared directly to the results of the present study. Concurrent to the first detection in Austria, *R. raoultii* was also found in the Czech Republic in 2016 and in Slovakia in 2021, where *R. raoultii* was also documented in *H. inermis* [35,36]. However, in addition to growing attention and awareness of the diseases caused by *Rickettsia*, this could also be a sign of the spread of *R. raoultii* in Europe. Infections of *R. raoultii* in humans show a wide variance of symptoms, and range from subclinical cases to severe disease [37]. While *R. raoultii* is responsible for disease patterns such as tick-borne lymphadenopathy (TIBOLA), *Dermacentor*-borne necrosis erythema and lymphadenopathy (DEBONEL), or scalp eschar and neck lymphadenopathy (SENLAT), *R. helvetica* appears to be less pathogenic [38]. However, there are case reports of spotted fever rickettsiosis (SFR) triggered by *R. helvetica* infection [39]. These rickettsiae were also detected in *I. ricinus* in this study. Furthermore, *R. monacensis* was detected in a pool of *I. ricinus* ticks. This species is also considered to be human pathogenic and was recently detected for the first time in Austria [27,40]. As already mentioned above, it seems that *Rickettsiae* could spread in Europe and gain importance for human medicine in the future. In addition, the presence of human pathogenic *Rickettsiae* could pose a threat to military dog handlers in Burgenland.

The sampling areas in the present study and the MWDTC were located in a region where *D. reticulatus* is known to be endemic, and *B. canis* was detected in 7% of *D. reticulatus* ticks [4,41]. While a prevalence of 4.2% for *B. canis* was found in the dog population of the MWDTC, no evidence of *B. canis* could be found in the vectors in this study. This may indicate that vector surveillance alone (i.e., without examining a susceptible population) may be of limited value, or that the number of *D. reticulatus* ticks collected in the present study was too low. *Babesia microti* was detected in *I. ricinus*. This is the second report in Austria [27] and the first detection in this part of Austria (Burgenland). *Babesia microti* is a zoonotic piroplasmid species and can lead to malaria-like symptoms and, especially in immunosuppressed individuals, can have a fatal course. Additionally, it tends to have a long persistence and plays a role in transmission via blood transfusion [42]. It is a potential threat to both dog handlers, during outdoor work and training, and civilians in the investigated area in the present study.

Three different sequence types were detected, which may indicate different *Bartonella* species or strains within the same species. Thirty-seven sequences (accession numbers OL690436–OL690472) were detected in total. Positive findings were found in all tick species. The population of *H. inermis* ticks had the highest prevalence of *Bartonella* sp. (62.50%). To the authors’ knowledge, and from a review of the available literature, there has been no detection of *Bartonella* DNA described in *H. inermis* ticks.

*Arsenophonus* sp. was detected in seven pools of *I. ricinus* ticks (three pools of adults and four pools of nymphs). *Arsenophonus nansoniae* is known to be an endosymbiont of the tick-parasitic wasp *Ixodiphagus hookeri* and has previously been detected in ticks [43]. However, these bacteria are not considered pathogenic and are, therefore, an incidental finding that could indicate a previous infection of the ticks with *I. hookeri.*

All eight mosquito species found are considered native to Austria [18]. Of the mosquito species found, *Ae. vexans, Cx. pipiens* s. l., *Coquillettidia richiardii*, and *Cx. modestus* are known to be competent vectors for *D. immitis*. Furthermore, *Ae. vexans* and *Cx. pipiens* s. l. are also known as competent vectors for *D. repens* [44,45,46]. While *D. repens* was detected in the military dog population in the context of haematological examination in 2016 [21], no evidence of dirofilariae was found in the mosquitoes tested in the present study. However, previous evidence of *D. repens* in mosquitoes in Austria is limited to the mosquito species *Anopheles maculipennis* s.l., *An. algeriensis*, and *An. plumbeus* [20,47], which were not sampled in the present study. A study at several locations in Austria also failed to detect dirofilariae in mosquito monitoring, while *D. immitis* was detected in several dogs at the same location (animal shelters). Additionally, keeping dogs outdoors overnight is likely to have a major impact on the risk of mosquito bites and, therefore, potential transmission [48]. However, all of these dogs were imported from abroad [49]. As a limitation in the abovementioned study, all dogs were treated prophylactically with milbemycin oxime, and thus did not show microfilaraemia. Nevertheless, negative results of vector screenings close to a host population indicate—but do not exclude—the absence of a certain vector-borne pathogen.

## 4. Materials and Methods

### 4.1. Ticks

#### Collection and Specification

**Figure 4:**
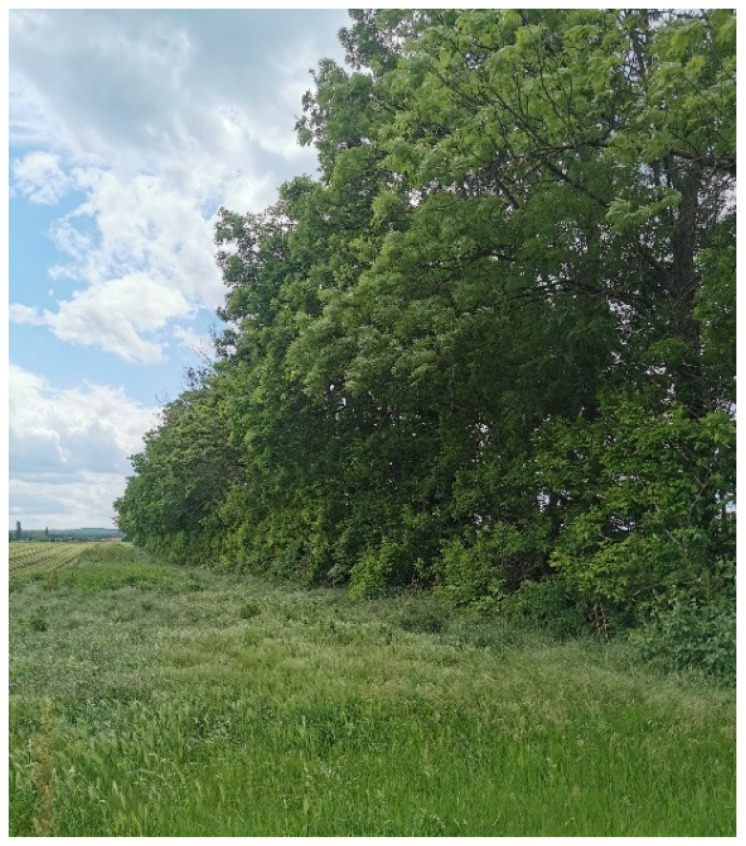
Shelterbelt, Burgenland, Austria. © Matthias Jüly, 2021.

The sampling sites were located next to the MWDTC in Kaisersteinbruch, Burgenland, Austria. The areas were selected according to the frequency of dog visits, suitable landscape, and accessibility. These criteria were determined by interviewing and observing the dog handlers. Finally, this resulted in three different locations for sampling. One location was in the forest, one was the dog run, and the third was a shelterbelt which was typical for this region (Figure 4. The sampling took place in spring 2019 and 2020 over a total of 12 days. The ticks were collected using a 50 cm × 100 cm flag made of cord fabric, fixed to a pole. The flag was turned every two meters, and the ticks were regularly removed from the flag with tweezers and stored in an Eppendorf tube (Eppendorf AG, Hamburg, Germany).

The collected ticks were morphologically examined at the Institute of Parasitology of the University of Veterinary Medicine in Vienna, and their species, sex, and developmental stage were determined. The examination was performed using a stereomicroscope (Olympus SZH10 Research Stereo; Olympus Austria, Vienna, Austria). Taxonomic determination was performed using existing keys [11,50]. Subsequently, adults (5 per pool) and nymphs (10 per pool) of *I. ricinus* were pooled according to species, sex, developmental stage, date, and sampling location for further processing. Ticks of the species *D. reticulatus*, *H. inermis*, and *H. concinna* were screened individually.

### 4.2. Mosquitoes

#### Collection and Differentiation

A CO_2_-baited mosquito trap (BG-Sentinel^®^, Biogents, Regensburg, Germany) was placed directly next to the dog kennels once a month for 24 h from May to September 2019. The mosquitoes were then pooled by month and species following published identification keys [51].

### 4.3. DNA Extraction and Pathogen Detection

#### 4.3.1. DNA Extraction

Prior to DNA extraction, ticks were rinsed first with tap water, then with 3% H_2_O_2_, then again with tap water (2x) with 70% ethanol, and finally with tap water, and dried on a paper towel. After washing, each pool was placed in a Tissue Lyser (Tissue Lyser II, Qiagen GmbH, Hilden, Germany) (at 30 bps) with 500 µL dH_2_O and a 3 mm tungsten carbide bead (Qiagen GmbH, Hilden, Germany) for 5 min. The supernatant was then used for DNA extraction. The extraction of the DNA of the ticks and mosquitoes was performed using the DNeasy^®^ Blood and Tissue kit (Qiagen GmbH, Hilden, Germany) according to the manufacturer’s protocol (Qiagen GmbH, Hilden, Germany).

#### 4.3.2. Pathogen Detection

Conventional PCR protocols for the detection of *Borrelia* spp., *Rickettsia* spp., *Bartonella* spp., Anaplasmataceae, and *Francisella tularensis* in ticks were employed. In addition, samples were screened by nested PCR targeting Piroplasmida (Table 2). Mosquitoes were screened for filarioid helminths using touchdown PCRs. The PCRs were carried out in a final volume of 25 μL using 5X Green Reaction Buffer and GoTaq^®^ G2 Polymerase (5 U/µL; Promega, Germany). The PCR products were analysed by electrophoresis on 1.8% agarose gels stained with Midori-Green Advance^®^ (Biozym, Hessisch Oldendorf, Germany) and subsequently sequenced (LGC Genomics GmbH, Berlin, Germany). For details on primers and protocols, see Table 2.

### 4.4. Statistics

Statistical analyses were performed using Microsoft Excel 2013 (Microsoft Corporation, Redmond, DC, USA). The Minimal Infection Rate (MIR) for the pooled *I. ricinus* ticks was calculated using the following formula [52]:

$$MIR=\frac{Number\;of\;positive\;pools}{Number\;of\;I.\;ricinus}\times 100$$

The maximum likelihood estimation (MLE) was calculated with the following formula [53].

MLE=1−(1−positive pools/number of pools)^1/size of pools

**Table 2:** PCR protocols for molecular analysis of the ticks and mosquitoes.

Organism	Locus	Primer Sequences	Amplification Protocol [Reference]	Product Size (bp)
Rickettsiae	Citrate Synthase	RpCS.877p: 5′-GGGGGCCTGCTCACGGCGG-3′ RpCS.1258n: 5′-ATTGCAAAAAGTACAGTGAACA-3	95 °C 5 min; 40×: 95 °C 30 s, 50 °C 30 s, 72 °C 1 min; 72 °C 10 min [54]	381
Anaplasmataceae	16s rRNA	EHR16SD_for: 5′-GGTACCYACAGAAGAAGTCC-3′ EHR16SR_rev: 5′-TAGCACATCATCGTTTACAGC-3	95 °C 2 min; 35×: 94 °C 1 min, 54 °C 30 s, 72 °C 30 s; 72 °C 5 min [55]	345
*Francisella tularensis*	17 kDa lipoprotein gene	TUL4-435: 5′-GCTGTATCATCATTTAATAAACTGCTG-3′ TUL4-863: 5′-TTGGGAAGCTTGTATCATGGCACT-3	94 °C 5 min; 40×: 94 °C 1 min, 51 °C 1 min, 72 °C 1 min; 72 °C 10 min [56]	400
Piroplasmida	18S rRNA	Nest 1: BTH-1F: 5′-CCTGAGAAACGGCTACCACATCT-3′ BTH-1R: 5′-TTGCGACCATACTCCCCCCA-3′ Nest 2: G-2_for: 5′-GTCTTGTAATTGGAATGATGG-3′ G-2_rev: 5′-CCAAAGACTTTGATTTCTCTC-3′	Nest 1: 94 °C 2 min; 40×: 95 °C 30 s, 68 °C 1 min, 72 °C 1 min; 72 °C 10 min Nest 2: 94 °C 2 min; 40×: 95 °C 30 s, 60 °C 1 min, 72 °C 1 min; 72 °C 10 min [57]	Nest 1: 700 Nest 2: 561
*Borrelia*	16S rRNA	Borr_allg_for: 5′-ACGCTGGCAGTGCGTCTTAA-3′ Borr_allg_rev: 5′-CTGATATCAACAGATTCCACCC-3′	94 °C 5 min; 40×: 94 °C 1.5 min, 63 °C 2 min, 72 °C 2 min; 72 °C 10 min [58]	674 bp
*Bartonella*	gltA	BhCS.781p: 5′-GGGGACCAGCTCATGGTGG-3′ BhCS.1137n: 5′-AATGCAAAAAGAACAGTAAACA-3′	94 °C 5 min; 40×: 94 °C 1 min, 54 °C 1 min, 72 °C 1 min; 72 °C 10 min [59]	379 bp
Filarioid helminths	COI	RpCS.877p: 5′-GGGGGCCTGCTCACGGCGG-3′ RpCS.1258n: 5′-ATTGCAAAAAGTACAGTGAACA-3	94 °C 2 min; 8×: 94 °C 45 s, 51 °C 45 s (reduced by 0.5 °C/cycle), 72 °C 1.5 min; 25×: 94° C 45 s, 45 °C 45 s, 72 °C 1.5 min; 72 °C 7 min [60]	688 bp

## 5. Conclusions

A number of potentially pathogenic microorganisms were detected in the ticks examined in the present study. Some of the pathogens found were detected in ticks in Austria for only the second time (*B. mijamotoi*, *B. lustinaniae*, and *B. microti*). A dog population from the MWDTC examined in parallel to vector collection displayed infections with *B. canis*, *D. repens*, and *B. burgdorferi* s. l. [21]. However, only *B. burgdorferi* s.l. was found in the corresponding vector surveillance. This divergence of results has implications for the interpretation of vector collection and examination for the surveillance of (emerging) diseases. Based on the presented results, vector surveillance alone does not seem to be sufficient and should be combined with the surveillance of an exposed population whenever possible.

The discovery of *H. inermis* as the second-largest subsample must be highlighted, since this species has not been detected in Austria for a long time and its vector competence is unclear. Further studies are needed to confirm or rule out this species as a significant vector.

In connection with the discovery of a number of veterinary and human pathogenic (zoonotic) infectious agents in eastern Austria, as well as the exposed working conditions of military dogs and dog handlers in this area, the current findings lead to the urgent recommendation of an awareness campaign, and urge an evaluation of protective measures for both dogs and their trainers. Further investigations of both civilian and military dog populations and vectors in this and other areas of Austria are strongly recommended.

## Figures and Tables

**Table 1:** Relative frequencies (as percentages) of the pathogens (in parentheses: Genbank^®^ accession numbers) in relation to the total population pools of the tick species (*I. ricinus* pools: 5 adults or 10 nymphs; the other species were tested individually).

Pathogen	*I. ricinus*	*D. reticulatus*	*H. inermis*	*H. concinna*
*Borrelia afzelii* (OM033637)	29.38	0.00	0.00	0.00
*Borrelia miyamotoi* (OM033638)	7.37	0.00	0.00	0.00
*Borrelia garinii* (OM033640, OM033643, OM033644)	6.32	0.00	0.00	0.00
*Borrelia burgdorferi* s. s. (OM033638, OM033645)	5.26	0.00	0.00	0.00
*Borrelia lustinaniae* (OM033641)	3.68	0.00	0.00	0.00
*Borrelia valesiana* (OM033642)	0.53	0.00	0.00	0.00
*Rickettsia helvetica* (OM039458)	51.05	0.00	0.00	0.00
*Rickettsia monacensis* (OM039462)	0.53	0.00	0.00	0.00
*Rickettsia raoultii* (OM039459)	0.00	60.98	0.00	0.00
*Rickettsia* sp. (OM039460, OM039461)	1.05	2.44	64.58	60.00
*Bartonella* spp. (OL690436–OL690472)	1.58	2.44	62.50	60.00
*Babesia microti* (OL960636)	0.53	0.00	0.00	0.00
*Arsenophonus* sp. (OM001650)	3.68	0.00	0.00	0.00

## Data Availability

Not applicable.
